# Does solar irradiation drive community assembly of vulture plumage microbiotas?

**DOI:** 10.1186/s42523-020-00043-7

**Published:** 2020-07-14

**Authors:** Gary R. Graves, Kenan O. Matterson, Christopher M. Milensky, Brian K. Schmidt, Michael J. V. O’Mahoney, Sergei V. Drovetski

**Affiliations:** 1 Department of Vertebrate Zoology, National Museum of Natural History, Smithsonian Institution, Washington, DC, 20013 USA; 2 Center for Macroecology, Evolution, and Climate, Globe Institute, University of Copenhagen, DK-2100 Copenhagen Ø, Denmark; 3 Department of Biological, Geological and Environmental Sciences (BiGeA), University of Bologna, 48100 Ravenna, Italy; 4 Department of Invertebrate Zoology, National Museum of Natural History, Smithsonian Institution, Washington, DC, 20013 USA; 5 Laboratories of Analytical Biology, National Museum of Natural History, Smithsonian Institution, Washington, DC, 20013 USA; 6 Current address: USGS Patuxent Wildlife Research Center, 10300 Baltimore Avenue, BARC-East Bldg. 308, Beltsville, MD 20705 USA

**Keywords:** Cathartiformes, *Deinococcus*, Extremophile, Keratin-degrading bacteria, Melanized plumage, New World vultures, Plumage microbiota, Solar irradiation, Spread-wing sunning, Ultraviolet (UV) light

## Abstract

**Background:**

Stereotyped sunning behaviour in birds has been hypothesized to inhibit keratin-degrading bacteria but there is little evidence that solar irradiation affects community assembly and abundance of plumage microbiota. The monophyletic New World vultures (Cathartiformes) are renowned for scavenging vertebrate carrion, spread-wing sunning at roosts, and thermal soaring. Few avian species experience greater exposure to solar irradiation. We used 16S rRNA sequencing to investigate the plumage microbiota of wild individuals of five sympatric species of vultures in Guyana.

**Results:**

The exceptionally diverse plumage microbiotas (631 genera of Bacteria and Archaea) were numerically dominated by bacterial genera resistant to ultraviolet (UV) light, desiccation, and high ambient temperatures, and genera known for forming desiccation-resistant endospores (phylum Firmicutes, order Clostridiales). The extremophile genera *Deinococcus* (phylum Deinococcus-Thermus) and *Hymenobacter* (phylum, Bacteroidetes), rare in vertebrate gut microbiotas, accounted for 9.1% of 2.7 million sequences (CSS normalized and log_2_ transformed). Five bacterial genera known to exhibit strong keratinolytic capacities in vitro (*Bacillus, Enterococcus, Pseudomonas, Staphylococcus,* and *Streptomyces*) were less abundant (totaling 4%) in vulture plumage.

**Conclusions:**

Bacterial rank-abundance profiles from melanized vulture plumage have no known analog in the integumentary systems of terrestrial vertebrates. The prominence of UV-resistant extremophiles suggests that solar irradiation may play a significant role in the assembly of vulture plumage microbiotas. Our results highlight the need for controlled in vivo experiments to test the effects of UV on microbial communities of avian plumage.

## Background

Avian evolution has been shaped by millions of years of natural selection by bacteria, archaea, fungi, and metazoan ectoparasites [1–6] that digest or degrade the keratinous feathers, nails, and foot, leg, and bill coverings of birds [7–9]. Feathers are largely composed of polymeric filaments of *β*-keratin that are tightly bound to an amorphous polymer matrix. Once grown, feathers are metabolically inert and have no vascular connection to the body. The bulk of microbial research on avian plumage has concentrated on the industrial potential of keratinolytic bacteria isolated from soil and commercial poultry waste to convert feathers [8, 9] into animal feed [10–16]. Microbial keratinases target cross-linked structural peptides that make feather keratin insoluble [15]. The continued focus on domestic poultry and keratinolytic bacteria has resulted in a substantial void in our knowledge of the taxonomic diversity, host specificity, and assembly of microbial communities in the plumage of the 10,135 wild bird species [5, 6, 17–28].

Birds have evolved a suite of behaviours such as sunning, dusting, and anting to maintain plumage quality [29–31]. Avian sunning is phylogenetically and geographically widespread and serves a multiplicity of purposes [29, 32, 33]. Sunning birds typically spread their wings and tail for maximal exposure to the sun [29, 33]. Heat absorption and thermoregulation are the most frequently hypothesized functions [29, 33–35], but sunning is often performed at thermoneutral temperatures in tropical and temperate latitudes. In fact, birds may voluntarily sunbathe to the brink of hyperthermia [29, 36], indicating some purpose other than regulation of body temperature. Other traditional explanations for sunning include plumage-drying [29, 35], inhibition of feather-chewing lice [30, 36], restoration of feather shape [37], moult facilitation [38], production of vitamin D [33], and preen gland stimulation [33]. After 75 years of study, researchers have been unable to reach consensus on a primary driver that comfortably explains the occurrence of sunning in more than 50 taxonomic families and 21 orders of birds [29, 33].

The detection of keratinolytic bacteria in the plumage of a wide variety of wild bird species [5, 6] led to a new hypothesis for the function of avian sunning behavior [39]. Saranathan and Burtt [39] studied the effect of sunlight (280–750 nm) on *Bacillus licheniformis*, a common soil-dwelling keratinolytic bacterium found widely on avian plumage [5]. Based on in vitro experiments, they posited that sunning might regulate potentially harmful plumage microorganisms. Their hypothesis has failed to gain traction and there is little empirical evidence that solar irradiation alters the assembly and abundance of plumage microbiotas in wild birds. Recent studies, however, suggest that feather structure and pigmentation may affect ultraviolet (UV) reflectance and absorbance of solar radiation and may thus affect bacterial diversity and abundance [40, 41].

**Fig. 1:**
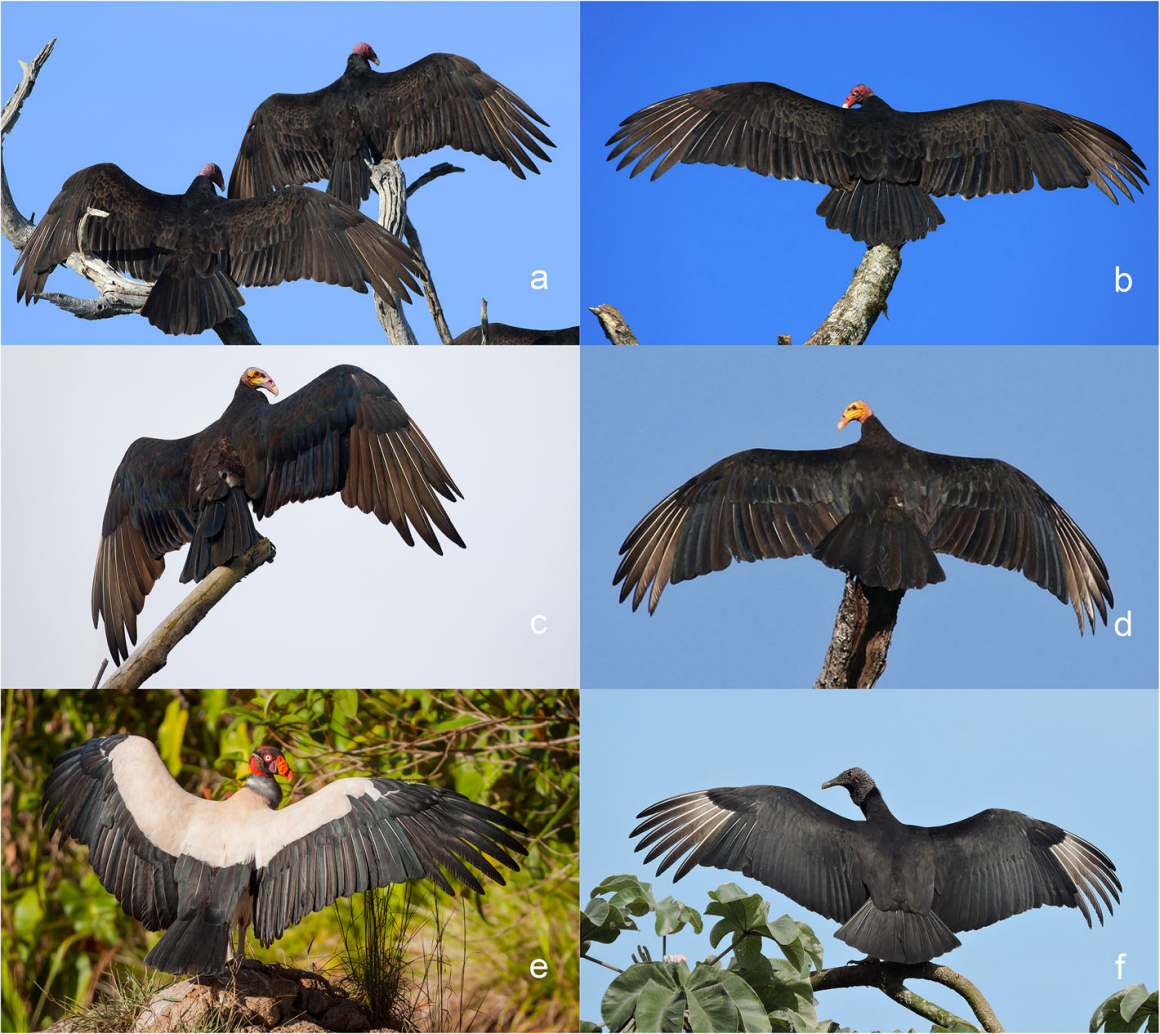
Spread-wing sunning in New World vultures (Cathartiformes). **a-b** turkey vulture (*Catharates aura*). **c** greater yellow-headed vulture (*Cathartes melambrotos*). **d** lesser yellow-headed vulture (*Cathartes burrovianus*). **e** king vulture (*Sarcoramphus papa*). **f** black vulture (*Coragyps atratus*). Photo credits: **a**, Gary R. Graves; **b**, Brian K. Schmidt; **c**, Esteban Argerich; **d**, Charley Hesse; **e**, Yeray Seminario; **f**, Francisco Dubón

Bird plumage exposed to intense solar radiation constitutes one of the more extreme microbial environments found on or inside terrestrial and aquatic animals. For example, maximum plumage temperatures recorded in sunning brown-necked ravens (*Corvus ruficollis*) ranged from 49.2 °C at skin level to an extraordinary 83.9 °C at feather surfaces [42]. Black plumage attains significantly higher temperatures than pale plumage in direct sun at low wind speeds [36, 42, 43]. The disproportionate number of bird species with black or dark gray plumage in arid regions [43] suggests that comparably high plumage temperatures are routine. Hundreds of diurnal bird species live in open-habitat formations or forage aerially in full sunlight, but few species experience as much solar irradiation as New World vultures [44], a monophyletic group (Cathartiformes) of obligate scavengers of vertebrate carrion [44–46]. Vultures engage in thermal soaring [47] and are renowned for conspicuous spread-wing sunning at roosts and loafing sites [35, 47]. Indeed, sunning appears to be a deeply-ingrained behaviour in the Cathartiformes. Sunning vultures perch upright and typically face directly toward or away from the sun to maximize the incidence of sunlight on their plumage (Fig. 1). Both dorsal and ventral surfaces of flight feathers are exposed to direct solar irradiation during spread-wing sunning as individuals shift positions. Ventral and dorsal contour (body) plumage also receives direct solar irradiation during sunning bouts.

We used 16S rRNA sequencing to investigate the taxonomic diversity and abundance of plumage microbiota in five species of sympatric vultures in Guyana: *Sarcoramphus papa* (king vulture), *Coragyps atratus* (black vulture), *Cathartes aura* (turkey vulture), *Cathartes burrovianus* (lesser yellow-headed vulture), and *Cathartes melambrotos* (greater yellow-headed vulture). We predicted that vulture plumage microbiotas would be exceptionally rich based on their natural history and the myriad potential transmission mechanisms. The downy plumage of nestling vultures is exposed to soil microbiota at terrestrial nest sites and carrion regurgitated by adults [47]. Vertical transmission from adults to nestlings is enabled during brooding and feeding. In adults, horizontal transmission of plumage microbiota is facilitated by mating and allopreening behaviour [47, 48]. Adult plumage, facial skin, and legs and feet are fouled by the rich microbiotas of decomposing flesh and body fluids of vertebrate carrion [49]. The habit of inserting their heads into body cavities undoubtedly explains the surprisingly diverse microbiotas of vulture facial skin [50]. Intraspecific and interspecific transmission almost certainly takes place when multispecies feeding aggregations jostle for access to carcasses. Vulture plumage is also chronically exposed to soil and dust kicked up at feeding sites, transferred from feet to plumage during preening, and when vultures rub their plumage on soil [48]. Adding to the long list of contamination routes, plumage microbiotas are inoculated by falling excrement at communal roosting sties and aerial particulates during flight. In sum, vulture plumage represents a model system for investigating the consequences of solar irradiation on rich microbial communities.

## Results

### Vulture plumage hosts diverse microbial assemblages

We sequenced the hypervariable V4-V5 region of the 16S SSU rRNA gene isolated from dorsal and ventral feather tracts of 34 field-collected specimens (see Methods). We obtained 790 to 101,187 (median = 34,441) filtered sequences per plumage sample and a total of 2,722,291 sequences in the pooled dataset (Additional file 1). Only a single plumage sample was represented by fewer than 2000 sequences. We emphasize generic-level comparisons in our analyses because a majority of the amplicon sequence variants (ASVs) were unclassified at the species level in the SILVA database [51]. We present cumulative sum scaled [52] and log_2_-transformed ASV normalized sequence counts (hereafter log_2_ CSS) to account for variation in sequencing depth among samples [53] and non-normal distribution of abundances among ASVs.

**Fig. 2:**
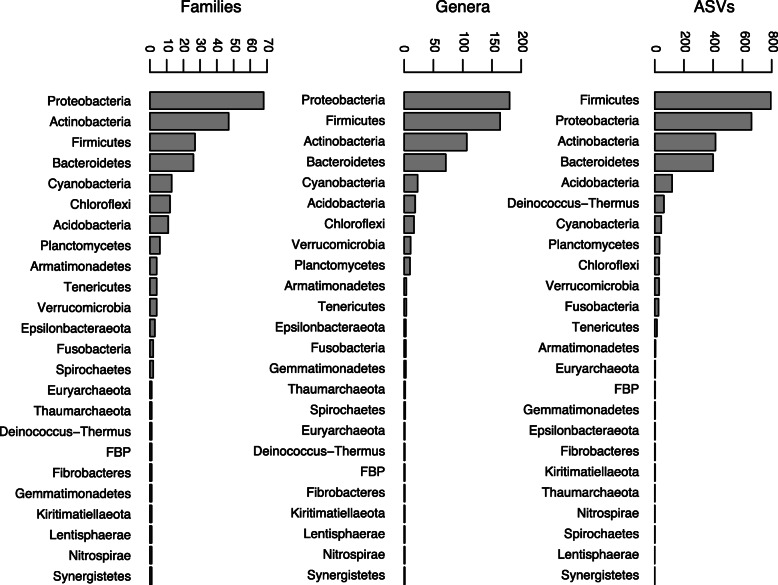
Rank-abundance of microbial taxa by phylum in New World vultures. Number of bacterial and archaeal ASVs, genera, and families by phylum observed in the pooled data set of New World vulture plumage samples

The filtered data contained 2670 ASVs, 631 genera, 239 families, 118 orders, 49 classes, and 24 phyla of Bacteria and Archaea (Additional files 2,3). Rarefaction curves for ASVs and microbial genera, families, and phyla reached asymptotes between 1000 and 2000 sequences per sample (Additional file 4). The majority of ASVs (85.4%) originated from four bacterial phyla: Firmicutes (29.7%), Proteobacteria (24.8%), Actinobacteria (15.6%), and Bacteroidetes (14.9%). Rank-abundance histograms of ASVs, genera, and families by phylum were strongly left-skewed (Fig. 2). The rarest 10 bacterial phyla contributed only 1.1% of the ASVs. Archaea (phyla Euryarchaeota and Thaumarchaeota) constituted a mere 0.2% of the ASVs. The plumage microbiota of the pooled sample of vulture species included 85 bacterial genera, 21 families, five orders, and two classes that were taxonomically unclassified in the SILVA [51] database (Additional file 1).

#### Vultures exhibit subtle species-specific differences in microbial assemblages

**Fig. 3:**
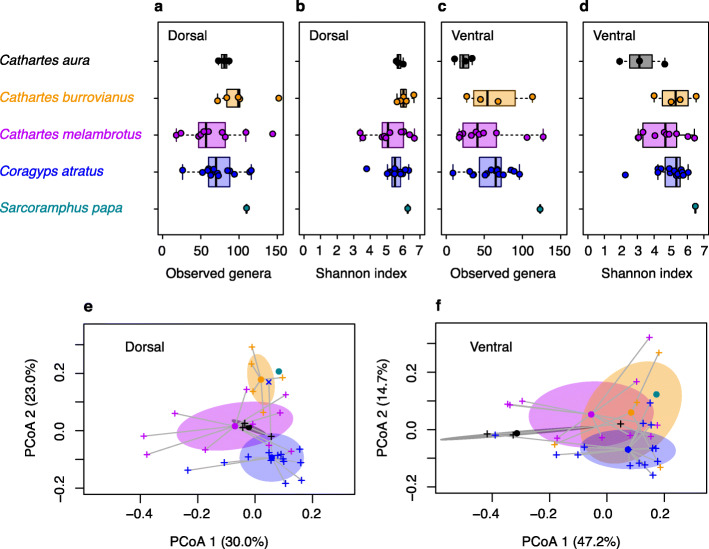
Microbial diversity metrics for vulture plumage. **a-d** Box plots of number of microbial genera and Shannon-Wiener diversity indices observed for dorsal and ventral plumage tract samples of five vulture species. **e** Principal coordinate analysis (PCoA) plots of microbial genera for dorsal plumage tract samples were based on weighted UniFrac distances. Data for vulture species are colour coded. Crosses represent vulture plumage samples and circles indicate centroids for vulture species. Shaded areas around centroids indicate 68% confidence intervals (one standard deviation). **f** PCoA plots for ventral plumage tract samples

The observed number of microbial genera ranged from 9 to 152 per plumage sample, averaging 77.6 and 56.2 genera for dorsal and ventral plumage samples, respectively (Fig. 3a,c). Shannon-Wiener diversity indices exhibited a wide range of values (Fig. 3b,d). Principal coordinate analysis (PCoA) based on weighted UniFrac distances revealed only modest separation of dorsal plumage microbial communities of *Coragyps atratus* from those of the three species of *Cathartes* (Fig. 3e,f). The PCoA plot for ventral plumage displayed more spatial overlap, reflecting extensive sharing of dominant microbial ASVs. Between-group weighted UniFrac distances were significant for vulture species (PERMANOVA *F*_4,56_ = 3.78, *R*^2^ = 0.20, *P* < 0.001), marginally significant for plumage tracts (*F*_1,56_ = 2.51, *R*^2^ = 0.03, *P* = 0.04), but were non-significant for the interaction, vulture species × plumage tract (*F*_4,56_ = 0.90, *R*^2^ = 0.05, *P* = 0.57).

#### Extremophile bacteria are abundant in heavily melanized plumage

**Fig. 4:**
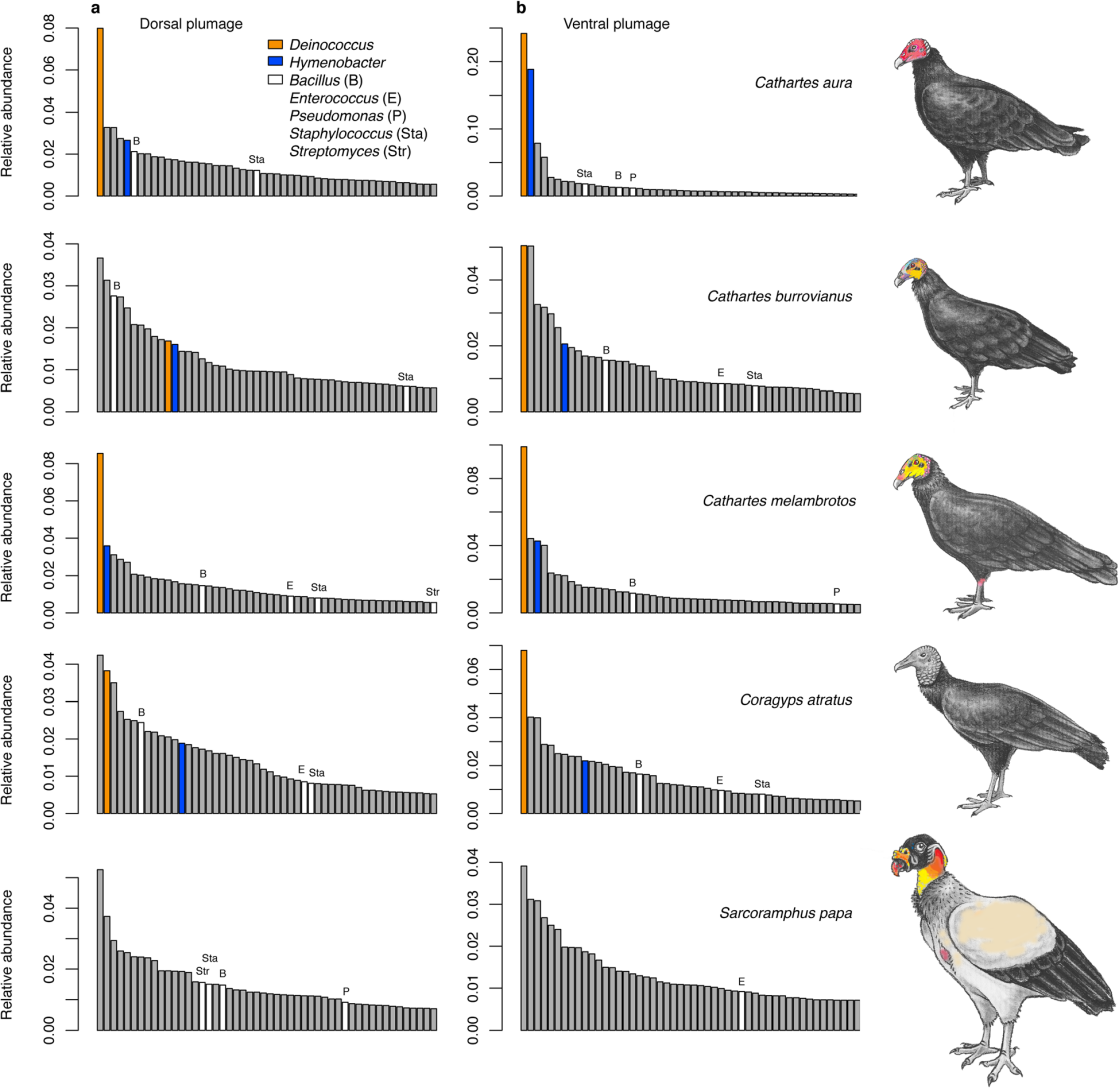
Ranked abundance of bacterial genera. **a** Dorsal plumage tract. **b** Ventral plumage tract. Histograms are limited to the 50 most abundant bacterial genera for each plumage tract within species. The extremophiles, *Deinococcus* and *Hymenobacter*, are denoted by orange and blue bars, respectively. Genera known from plumage of wild birds that exhibit keratinolytic activity in vitro are denoted in white. Initials signify *Bacillus* (B), *Enterococcus* (E), *Staphylococcus* (Sta), Streptomyces (Str), and *Pseudomonas* (P). No archaeal genera occurred among the top 50. The y-axis represents proportional abundance (log_2_ CSS normalized counts for each genera divided by the summed log_2_ CSS counts for all genera observed across individuals within a species). Illustrations of vulture species are proportionally sized and were modified from original drawings by Megan K. Viera

Rank-abundance plots of microbial genera were strongly left-skewed for dorsal and ventral plumage tracts (Fig. 4). Perhaps the most surprising finding of the 16S rRNA survey was the marked abundance of extremophiles in heavily-melanized plumage of *Coragyps atratus* and the three species of *Cathartes*. The genus *Deinococcus* (phylum Deinococcus-Thermus), an aerobic polyextremophile that is remarkably resistant to ionizing radiation, UV, and desiccation [54–57], constituted 6.3% of the log_2_ CSS normalized counts of filtered sequences in the pooled sample (Additional file 3). It was 1.7 × more abundant than the second most common genus, *Clostridium* sensu stricto 1 (phylum Firmicutes), which is commonly found in the vulture gut [50]. The third most abundant genus, *Hymenobacter* (phylum Bacteriodetes) also exhibits extremophile traits [58].

Collectively, *Deinococcus* and *Hymenobacter* (of 631 genera) constituted 9.1% of the log_2_ CSS normalized counts of filtered sequences recovered from black plumage of *Coragyps atratus* and the three species of *Cathartes*. By comparison, *Deinococcus* was rare (rank-abundance <60th) and *Hymenobacter* was absent in the beige plumage of the sole specimen of *Sarcoramphus papa*. Neither *Deinococcus* nor *Hymenobacter* is known to be pathogenic in vertebrates [56, 58], nor have these extremophiles been identified as keratinolytic agents in wild bird populations [5, 18, 21, 39]. *Deinococcus* has been identified as a possible contaminant of laboratory reagents and DNA extraction kits [59]. We ruled out contamination in our study because DNA was not detected in blank samples processed with the extraction and clean up kits. Additionally, only 5% of microbiota samples (*n =* 208) from other anatomical regions (e.g., facial swabs, esophagus, duodenum, colon) obtained from the same vulture specimens, and randomly interspersed with feather samples in the same Illumina MiSeq sequencing runs, yielded sequence classified as *Deinococcus* or *Hymenobacter* (unpublished data). Moreover, only 5 of 64 (non-plumage samples) represented by low DNA concentrations (< 1 ηg/μL) contained *Deinococcus* or *Hymenobacter.* High standardized sequence counts of *Deinococcus* and *Hymenobacter* were exclusive to melanized plumage samples of *Coragpys atratus* and the three *Cathartes* species.

Bacterial genera capable of forming desiccation-resistant endospores (phylum Firmicutes, order Clostridiales) were common in both black and beige vulture plumage. Genera which exhibit keratinolytic activity in vitro (*Bacillus, Enterococcus, Pseudomonas, Staphylococcus, Streptomyces*), and are known to occur in the plumage of wild birds [5, 18, 21, 39], collectively constituted only 4.0% of the CSS log2 normalized sequence counts in vulture plumage (Fig. 4, Additional file 3).

**Fig. 5:**
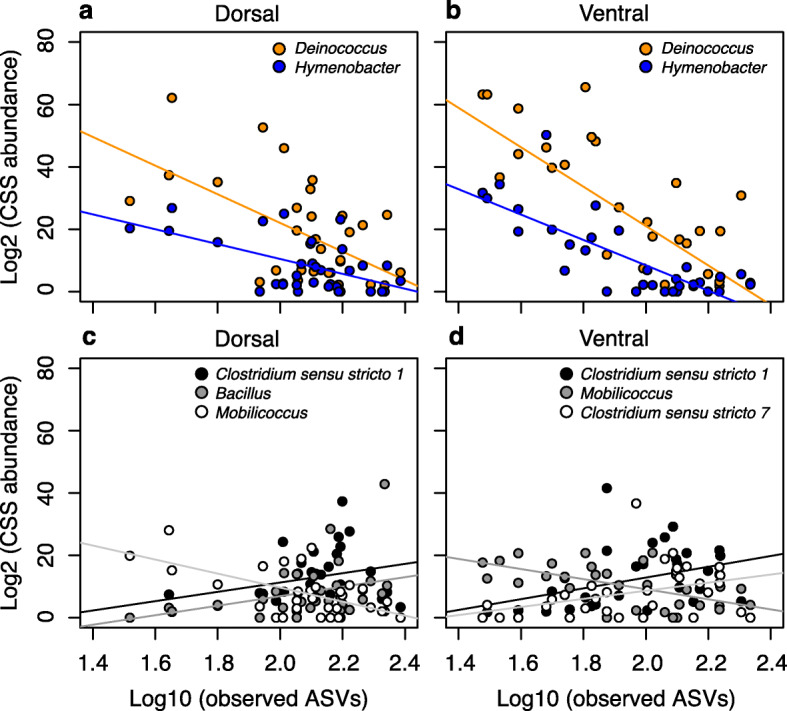
Abundance of the commonest bacterial genera. Scatterplots of CSS log_2_ normalized counts of the commonest bacterial genera and the number of ASVs observed in plumage samples. **a***Deinococcus* (adjusted *R*^*2*^ = 0.30, *P =* 0.0006) and *Hymenobacter* (adjusted *R*^*2*^ = 0.30, *P =* 0.0001) in dorsal plumage samples. **b** Comparable scatterplots for ventral plumage: *Deinococcus* (adjusted *R*^*2*^ = 0.52, *P =* 0.000001) and *Hymenobacter* (adjusted *R*^*2*^ = 0.59, *P =* 0.0000001). **c** Scatterplots of the second (*Clostridium* sensu stricto 1; adjusted *R*^*2*^ = 0.10, *P =* 0.043), fourth (*Bacillus*; adjusted *R*^*2*^ = 0.10, *P =* 0.041), and fifth most abundant genus (*Mobilicoccus*; adjusted *R*^*2*^ = 0.35, *P =* 0.0001) in dorsal plumage samples. **d** Comparable scatterplots for ventral plumage: *Clostridium* sensu stricto 1 (adjusted *R*^*2*^ = 0.16, *P =* 0.012), *Mobilicoccus* (adjusted *R*^*2*^ = 0.33, *P =* 0.0003), and *Clostridium* sensu stricto 7 (adjusted *R*^*2*^ = 0.13, *P =* 0.024). The x-axis represents the number of ASVs (log_10_) observed in individual samples. *R*^*2*^ and two-tailed *P-*values were derived from simple linear regression

The five most abundant microbial genera comprised 17.5% of the CSS log2 normalized counts in the pooled sample of vultures (Fig. 5). Abundances of the extremophile genera *Deinococcus* and *Hymenobacter* (Fig. 5a,b) were strongly negatively correlated with ASV richness in dorsal and ventral plumage tracts. The abundance of three other common bacterial genera (*Clostridium* sensu stricto 1, *Mobilicoccus, Clostridium* sensu stricto 7) exhibited weak correlations with ASV richness (Fig. 5c,d).

## Discussion

Vulture plumage is exposed to a rich source pool of bacteria and archaea in carrion, soil, gut contents, aerial particulates, and other environmental sources [47]. The majority of bacterial genera detected in vulture plumage are drawn from phyla that dominate avian gut microbiotas (Firmicutes, Proteobacteria, Actinobacteria, Bacteroidetes) [60–63]. The Chao1 [64] estimate of generic richness in the combined dorsal and ventral plumage microbiotas was 956 genera (95% confidence interval, 858–1098 genera). A significant fraction (13.5%) of the 631 bacterial and archaeal genera detected in the 16S rRNA survey were taxonomically unclassified in SILVA database [51]. More than 70% of the bacterial and archaeal genera detected in our analyses have not been previously reported in the associated tables and appendices of published papers that investigated plumage microbiota with 16S rRNA sequencing [5, 6, 17–24, 27]. Vulture plumage microbiotas can accurately be described as hyper-diverse and taxonomically cryptic.

Non-experimental data must be interpreted cautiously, but we infer the comparative abundance of UV-resistant genera *Deinococcus* and *Hymenobacter* in plumage microbiotas as a signature of solar irradiation. *Deinococcus* is environmentally widespread but relatively rare and patchily distributed in media ranging from soil, dung, and sewage to stratospheric dust [56, 57]. The prevalence of *Deinococcus* in gut and respiratory tract microbiotas of vertebrates is poorly understood but it appears to occur at low frequencies [50, 60–63, 65, 66], if it was detected at all. Higher abundances have been noted in the gut of bats [67] and crocodile lizards [68]. Previous reports from avian plumage have been limited to trace occurrences in larks [24]. *Hymenobacter* has been isolated from desert soil, airborne dust, glacial ice, irradiated meat, dung, and water [58]. It is a rare component of vertebrate gut microbiotas [67] and the sole record in avian plumage was reported from black-plumaged swifts [22] that forage aerially and often fly nonstop for months [69].

The inverse correlations between the abundance of *Deinococcus* and *Hymenobacter* and ASV richness (Fig. 5) in vulture plumage microbiotas suggest that the combination of abiotic factors (UV radiation, heat, and desiccation) that suppress overall ASV richness, promotes the colonization, persistence, and growth of extremophiles. Agonistic interactions among ASVs waged through the production of bacteriocins [27, 70] or ASV competition for plumage-related resources such as preen-oil, dust, carrion smears, and feather keratin, may also contribute to the inverse relationships. We cannot directly address the underlying causes of the observed patterns because we lack data on the bacteriocins and nutritive constituents of plumage samples and the environmental conditions to which plumage was exposed in the months leading up to the collection date. The numerical dominance of *Deinococcus* and *Hymenobacter* in melanized vulture plumage has no known analog in the integumentary systems of terrestrial vertebrates. We note, however, that these bacterial genera are common constituents of biofilms recovered from solar panels [71, 72]. In any event, the unusual microbial rank-abundance profiles of vulture plumage appear to exhibit evidence of taxonomic filtering driven by solar irradiation.

All modern birds undergo periodic moult to replace worn, degraded, and damaged feathers [5, 7, 21] and the vast majority of species undergo at least one complete annual moult [73–76]. Bacterial degradation of feather keratin decreases the physical integrity of feathers, the functionality of plumage, and reduces the overall fitness of individuals. Moult reduces the load, at least temporarily, of bacteria and fungi that degrade plumage [5]. Replacement of flight feathers (primaries and secondaries) in New World vultures requires as long as 2 years to complete [74, 77]. The prolonged staggered moult in vultures imposes strong selection on maintenance behaviours that preserve the aerodynamic integrity of flight feathers necessary for efficient soaring. We hypothesize that spread-wing sunning in the Cathartiformes [35, 47] evolved to maximize solar irradiation on ventral and dorsal surfaces of flight feathers—noting that dorsal and ventral contour plumage is also irradiated in the process. Frequent exposure to UV radiation, and associated desiccation and heat, is likely to kill, inhibit growth, or induce sporulation in mesophilic and UV-sensitive bacteria and fungi, including many that degrade keratin.

Spread-wing sunning in vultures is just one of many postural sunning variants observed in birds [29, 33]. The occurrence of stereotyped sunning in more than 50 avian families and 21 avian orders suggests an ancient origin dating to the early Palaeogene when most avian orders arose [45, 46]. This raises the question of when the arms race between keratinolytic bacteria and the bearers of pennaceous feathers arose, noting that well-feathered *Archaeopteryx* and maniraptoriform dinosaurs appeared in the fossil record in the late Jurassic [78]. We forecast that intensive metagenomic surveys of plumage microbiotas of wild bird species will lead to novel insights on the evolution of pennaceous feathers in birds and dinosaurs.

## Conclusions

Our results offers tangible support for the hypothesis [39] that the principal function of avian sunning behavior is the regulation of bacteria and other keratinolytic agents that degrade plumage quality. The prominence of UV-resistant extremophiles in generic rank-abundance curves suggests that solar irradiation may play a significant role in the assembly of vulture plumage microbiotas. Indeed, the high abundance of UV-resistant extremophiles in avian plumage may be a signature of taxonomic filtering via solar irradiation. In any event, bacterial rank-abundance profiles of melanized vulture plumage have no known analog in the integumentary systems of terrestrial vertebrates. Our results highlight the need for controlled in vivo experiments to test the effects of UV on microbial communities of avian plumage.

## Methods

### New World vultures (Cathartiformes)

The avian order Cathartiformes forms a monophylytic lineage composed of seven living species of obligate carrion scavengers that are now geographically restricted to the Americas [44, 47]. New World vultures are often confused with the ecologically convergent Old World vultures (Accipitriformes), which have been decimated by livestock pharmaceuticals ingested in carrion in Africa and Eurasia [79, 80]. The most recent common ancestor of the New World vultures and Old World vultures (Accipitriformes) lived 55–65 million year bp [44–46]. Five New World vulture species occur in Guyana in northern South America (Figs. 1,4): *Sarcoramphus papa* (body mass 3.1 kg, wingspan 167 cm), *Coragyps atratus* (body mass 1.4–1.9 kg, wingspan 123–136 cm), *Cathartes aura* [81] (body mass 1.2–1.5 kg, wingspan 153–159 cm), *Cathartes melambrotos* (body mass 1.3–1.8 kg, wingspan 153–172 cm), and *Cathartes burrovianus* (body mass 0.8–0.9 kg, wingspan 144–156 cm).

### Collection localities

Vultures were sampled at two sites in southern Guyana in Upper Takuto-Upper Essequibo (Region 9), an area of exceptionally low human population density (0.42/km^2^).

### Site 1

Dadanawa Ranch (2° 49.28′ N, 59° 31.34′ W, 127 m above sea level). Thousands of free-range cattle graze a mosaic of unmanaged grassland and scrubby savannah on one of the largest ranches (> 4400 km^2^) in the Americas. Livestock mortality supports a large resident population of *Coragyps atratus.* More than 1500 vultures seasonly frequent a roost near the ranch headquarters. Smaller numbers of *Cathartes burrovianus* and *Cathartes aura* were observed daily. All three species were observed at baited net traps and carrion in multi-species feeding aggregations. *Sarcoramphus papa* is seasonally present but was not observed at the ranch headquarters during the brief sampling period. *Cathartes melambrotos* is unknown from open savannah grasslands on the ranch but occurs in adjacent blocks of forest.

### Site 2

Kanuku Mountains (3° 12.20′ N, 59° 24.20′ W, 109 m above sea level). The landcover consists of riverine forest and primary lower montane rainforest flanking sandbars of the Rupununi River. Little or no domestic livestock or agriculture occurs within 10 km of the study site. *Cathartes melambrotos* is common and outnumbers *Coragyps atratus, Cathartes aura,* and *Sarcoramphus papa. Cathartes burrovianus* is not known to occur in the forested interior of the Kanuku Mountains.

### Plumage sampling

The collecting period (16–29 October 2015) was timed for the non-breeding season during the final weeks of the pronounced fall dry season in southern Guyana. No measurable rainfall was recorded during the 50 day period prior to collecting at Dadanawa. Daily low and high temperatures, respectively, averaged 25.6 C and 35.6 C during the collecting period. Vultures were netted or shot and necropsied soon after death (usually 15–30 min) by gloved preparators [81–83]. Clusters of contour plumage were plucked with sanitized forceps from the center of the mantle (dorsal feather tract) and center of the upper breast (ventral feather tract), placed in cryovials, and frozen immediately in liquid nitrogen. In contrast to the large flight feathers (primaries and secondaries) that are replaced in a staggered moult that may require up to 2 years to complete [74, 77], the smaller contour feathers of the mantle and breast are replaced annually [74]. Contour plumage samples from *Coragyps atratus* and the three species of *Cathartes* were heavily melanized (black) whereas those from *Sarcoramphus papa* were pale beige (Figs. 1,4). All voucher specimens were deposited in the National Museum of Natural History (USNM), Smithsonian Institution (Additional file 1). A total of 34 vulture specimens were sampled: *Sarcoramphus papa* (*n =* 1), *Coragyps atratus* (*n =* 15), *Cathartes aura* (*n =* 3), *Cathartes melambrotos* (*n* = 10), and *Cathartes burrovianus* (*n* = 5).

### Molecular procedures

DNA was extracted from plumage samples using the PowerSoil DNA Isolation Kit (Mo Bio Laboratories, Carlsbad, CA, USA) following manufacturer protocols. Extracted DNA was purified with the PowerClean Pro DNA Clean-Up Kit (Mo Bio Laboratories, Carlsbad, CA, USA) to remove PCR inhibitors. To check for potential contaminants from the extraction and clean-up kits, we processed an extraction control without any feather material alongside the plumage samples. We quantified DNA concentration with the 1X dsDNA High-Sensitivity Assay Kit on Qubit 4.0 (Thermo Fisher Scientific Waltham, MA, USA). For feather samples we used 2 μL of DNA extract. For the extraction control, we increased the volume of extract to 10 μL and observed no detectable contamination within the threshold of the assay (10 pg/μL). We amplified and sequenced the V4–V5 region of the 16S SSU rRNA gene (370 bp) from Bacteria and Archaea using primers 515FB and 926R [84], following procedures outlined in the Earth Microbiome Project 16S protocol [84, 85]. Detailed description of PCR conditions, library preparation, and sequencing on an Illumina MiSeq (Illumina, Inc., San Diego, CA, USA) are provided in Drovetski SV, O’Mahoney M, Ransome EJ, Matterson KO, Lim HC, Chesser RT and Graves GR [86]. This protocol was modified to use AMPure XP beads (50 μL of library + 40 μL of beads; Beckman Coulter, Inc., Life Sciences Division, Indianapolis, IN, USA) to clean the final pooled library and the 500 or 600-cycle MiSeq Reagent Kits v2 or v3, (Illumina, Inc., San Diego, CA, USA), respectively, to accommodate the larger amplicon size. Each PCR reaction included a negative control.

### Illumina data processing

Samples were distributed among three Illumina runs (two runs with MiSeq Reagent Kits v2 500 cycles and one with v3 600-cycles kit). Initially, raw FASTQ sequence reads for each Illumina run were demultiplexed in QIIME2 v. 2019.1 (qiime demux emp-paired) [87]. Due to poor quality of the majority of reverse sequences, we processed only forward sequences (qiime demux emp-single). DADA2 [88] was used for quality filtering and chimera removal (−-p-chimera-method consensus). All sequences were truncated to 250 bp. This fragment covers V4 region of the 16S SSU rRNA and was 2–4 bp shorter than amplicons produced with primers 515FB and 806R [84], which are frequently used in microbiota studies that follow the Earth Microbiome Project 16S protocol [84, 85]. We combined feature tables and representative sequences from all three runs. We assigned taxonomy using the SILVA [51]-based 16S classifier for region V4 of the 16S SSU rRNA gene (SILVA 132 99% OTUs from 515F/806R region of sequences available at https://docs.qiime2.org/2019.1/data-resources/). Following taxonomic assignment, we filtered out sequences classified as Eukaryota, Mitochondria, Chloroplast, and those that were not classified to phylum. We aligned sequences and constructed phylogenetic trees using the align-to-tree-mafft-fasttree pipeline in QIIME2. The resulting feature table was exported into biom format and converted to the tab-delimited text in QIIME v. 1.9.1–20,150,604 [89]. Taxonomy was downloaded in the comma-separated format, and the rooted tree was downloaded in the newik format. Taxonomy was added to the tab-delimited table in Excel 14.7.7 using VLOOKUP function and the table was converted back to the biom format in QIIME. Finally, we filtered out ASVs with abundance fractions < 0.001 in individual samples using script from https://gist.github.com/adamrp/7591573. Sequence abundance by ASV was cumulative sum scaled and log_2_-transformed ASV abundances in QIIME (normalize_table.py–a CSS). All statistical analyses were based on log_2_ CSS ASV normalized sequence counts.

### Data analyses

We constructed rarefaction plots with R package Vegan 2.4–4 [90] (Additional file 4) to ensure our sequencing coverage was sufficient. We used R version 3.3.3 (http://www.R-project.org) to generate linear regression, box, and column plots.

We used QIIME for α and β-diversity analyses. We calculated the number of observed ASVs and Shannon-Wiener diversity indices (alpha_diversity.py) to compare microbiota richness and evenness among vulture species and feather tracts. Microbial richness at different taxonomic levels was assessed in QIIME (summarize_taxa_through_plots.py or summarize_taxa.py). We calculated weighted UniFrac distances [91] among individual samples in QIIME (beta_diversity_through_plots.py). To compare microbiota composition between feather tracts and among vulture species, we generated principal coordinate analyses (PCoA) plots using the *betadisper* function in Vegan 2.4–4. Weighted UniFrac distances were used in the Permutational Multivariate Analysis of Variance (PERMANOVA) [92] implemented in Vegan 2.4–4 [90] to test for the effect of species, plumage region (dorsal or ventral plumage tract), and their interaction on microbial variation among samples.

## Supplementary information

**Additional file 1.** Metadata. Laboratory identification numbers of feather samples, museum catalog numbers for voucher specimens, DNA concentration, number of filtered sequence reads, amplicon sequence variants (ASV), and number of microbial genera and families.

**Additional file 2.** Amplicon sequence variants (ASV) identified in the plumage samples of New World vultures.

**Additional file 3.** Log_2_ CSS abundance of bacterial genera identified in plumage samples of New World vultures.

**Additional file 4.** Rarefaction curves for ASVs and microbial genera, families, and phyla.

## Data Availability

The raw sequences archive has been deposited to GenBank: BioProject PRJNA521985: New World Vulture feather microbiota.

## Abbreviations

ASV: Amplicon sequence variant
CSS: Cumulative sum scaled
PCoA: Principal coordinate analysis
PERMANOVA: Permutational multivariate analysis of variance
UV: Ultraviolet light
