# Supplementary material for: Does solar irradiation drive community assembly of vulture plumage microbiotas?
Source: Anim Microbiome. 2020 Jul 14;2:24. doi: 10.1186/s42523-020-00043-7 (PMC7807431; doi:10.1186/s42523-020-00043-7)
Supplement: Supplementary file 4 — Additional file 4. Rarefaction curves for ASVs and microbial genera, families, and phyla. [file 42523_2020_43_MOESM4_ESM.pdf]

Additional file 4

Does solar irradiation drive community assembly of vulture plumage microbiotas?

Gary R. Graves , Kenan O. Matterson, Christopher M. Milensky, Brian K. Schmidt, Michael J. V. O'Mahoney, and Sergei V. Drovetski

Rarefaction curves for ASVs and microbial genera, families, and phyla

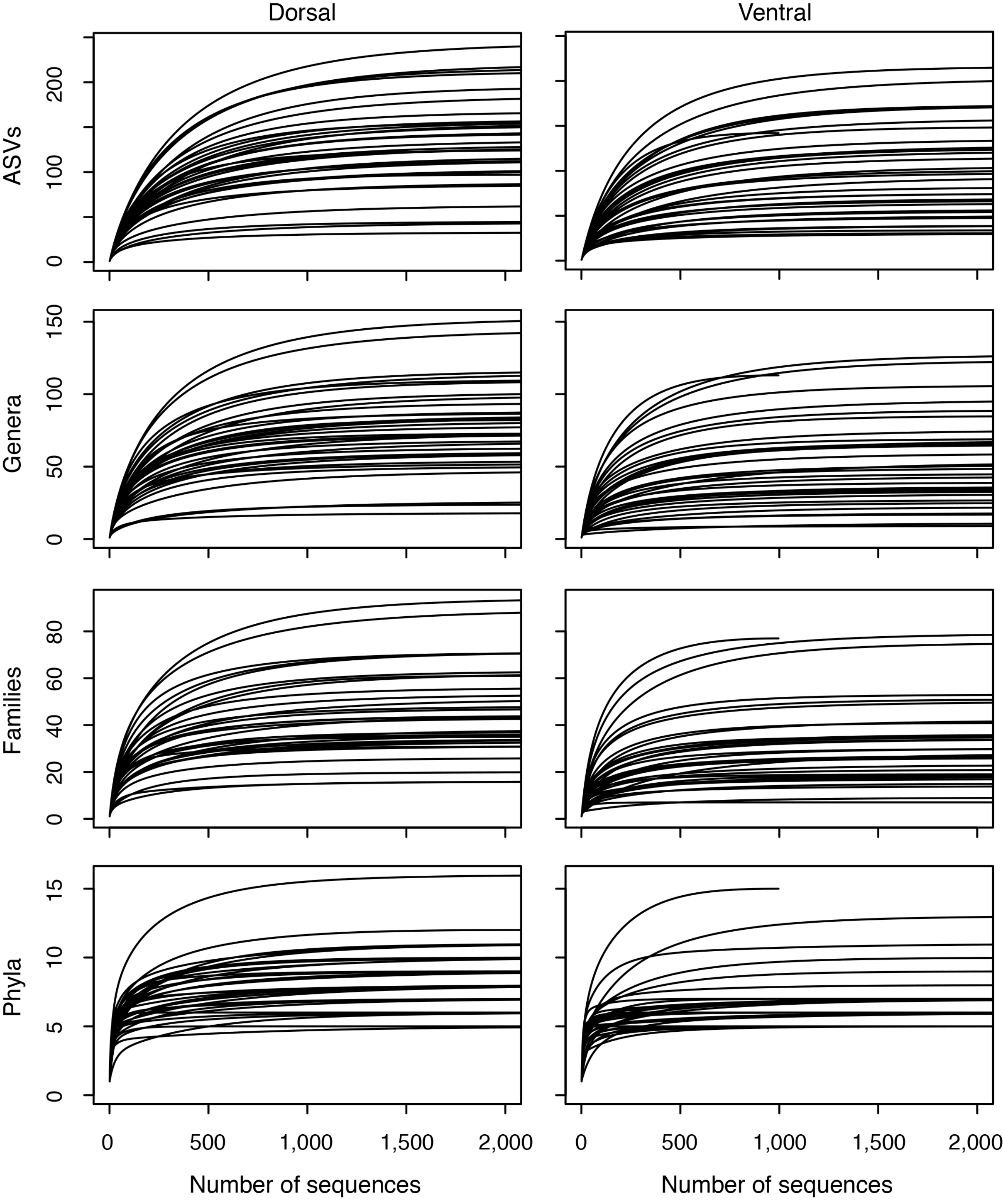
Dorsal
Ventral
ASVs
Genera
Families
Phyla
0
100
200
0
50
100
150
0
20
40
60
80
0
5
10
15
0
500
1,000
1,500
2,000
Number of sequences
Number of sequences
